# Modulating memristive performance of hexagonal WO_3_ nanowire by water-oxidized hydrogen ion implantation

**DOI:** 10.1038/srep32712

**Published:** 2016-09-07

**Authors:** Yong Zhou, Yuehua Peng, Yanling Yin, Fang Zhou, Chang Liu, Jing Ling, Le Lei, Weichang Zhou, Dongsheng Tang

**Affiliations:** 1 Synergetic Innovation Center for Quantum Effects and Application, Key Laboratory of Low-dimensional Quantum Structures and Quantum Control of Ministry of Education, College of Physics and Information Science, Hunan Normal University, Changsha, 410081, P.R. China

## Abstract

In a two-terminal Au/hexagonal WO_3_ nanowire/Au device, ions drifting or carriers self-trapping under external electrical field will modulate the Schottky barriers between the nanowire and electrodes, and then result in memristive effect. When there are water molecules adsorbed on the surface of WO_3_ nanowire, hydrogen ions will generate near the positively-charged electrode and transport in the condensed water film, which will enhance the memristive performance characterized by analogic resistive switching remarkably. When the bias voltage is swept repeatedly under high relative humidity level, hydrogen ions will accumulate on the surface and then implant into the lattice of the WO_3_ nanowire, which leads to a transition from semiconducting WO_3_ nanowire to metallic H_x_WO_3_ nanowire. This insulator-metal transition can be realized more easily after enough electron-hole pairs being excited by laser illumination. The concentration of hydrogen ions in H_x_WO_3_ nanowire will decrease when the device is exposed to oxygen atmosphere or the bias voltage is swept in atmosphere with low relative humidity. By modulating the concentration of hydrogen ions, conductive hydrogen tungsten bronze filament might form or rupture near electrodes when the polarity of applied voltage changes, which will endow the device with memristive performance characterized by digital resistive switching.

Tungsten trioxide (WO_3_) is probably the most thoroughly investigated representative of electrochromic and photochromic material^1^,^2^,^3^,^4^. Its electrochromic and photochromic properties offering significant potential applications in energy-efficient architecture, non-emissive information displays and smart window have been studied extensively^5^,^6^,^7^. Coloration of WO_3_ can be obtained, for example, by an electrochemical process (electrochromic effect) using an external voltage and a cation source, or by illumination with ultraviolet light (photochromic effect)^4^,^8^. The most well-known electrochromic technology is based on intercalation and de-intercalation of small ions in electrode materials. Moreover, WO_3_ is also a resistive switching material^9^,^10^,^11^,^12^. Resistive switching is another ion-modulated behavior, which has been found in many transition metal oxides, such as WO_3_^9^,^10^,^11^, NiO^12^, and TiO_2_^13^,^14^,^15^. It was proposed that the host metal cations or oxygen anions (or positively charged oxygen vacancies), as well as electrons, will drift under strong electrical field induced by external applied bias voltage. Metal cations drifting will lead to resistive switching by electrochemical metallization, while oxygen vacancies drifting will lead to resistive switching by valence change^16^,^17^,^18^,^19^,^20^,^21^,^22^. Therefore it is reasonable to conclude that the memristive performance of WO_3_ might be associated with its chromic properties, and then we can modulate the memristive performance by the methods for modulating the chromic properties.

It is the intriguing crystalline structures and electronic band structures of WO_3_ that endow it with outstanding physical properties and potential applications^23^,^24^,^25^,^26^,^27^,^28^,^29^. The higher atomic ratio of oxygen to tungsten leaves more empty interstices in the oxygen sub-lattice, and then WO_3_ can behave as a solid solvent towards accommodation of external species into its solid framework to form stable intercalation compounds, which endows WO_3_ with outstanding electronic and chromic properties. Hexagonal WO_3_ (h-WO_3_) is constructed from WO_6_ octahedra by sharing the equatorial oxygen, which forms one-dimensional hexagonal and trigonal tunnels along the c direction. Such a unique tunnel structure makes it easier for ions to transport in h-WO_3_^26^,^30^, and makes h-WO_3_ particularly useful in the application of electrochromic devices, rechargeable batteries, and memristors.

H-WO_3_ also displays peculiar electronic band structures. The electric potential energy of electrons at the bottom of the WO_3_ conduction band is less than the reduction potential energy of water, and electric potential energy of the holes at the top of the WO_3_ valence band is larger than the oxidation potential energy of water. Therefore, water molecules adsorbed on the surface of WO_3_ can be oxidized to produce hydrogen ions (H^+^ ions) and O_2_ by holes at the top of the valence band, while H^+^ ions cannot be reduced to produce H_2_ by electrons at the bottom of the conduction band^31^. Such electronic structures endow WO_3_ with photo-, electro- and thermo- chromic properties.

However, amorphous and polycrystalline WO_3_ films suffer from ion-trapping-induced degradation^1^. Ion intercalation and de-intercalation often cause transition of the crystalline phases. Moreover, electron scattering at grain boundary of the crystalline decreases the mobility of carriers in polycrystalline or amorphous films^32^. Meanwhile, the physical process of ion transport and then the memristor mechanism are still under debate, and the stability and repeatability of the existing memristors are relatively poor, which hinders memristor from being used in future device designs and circuit-level applications. Therefore single crystalline h-WO_3_ quasi-one-dimensional nanostructures might be an ideal platform for studying the effect of ion drifting on the electrical transport properties and exploring new methods to modulate the memristive performance.

In this paper, we report the effect of H_2_O adsorption on the memristive performance of the two-terminal Au/h-WO_3_ nanowire/Au device. H_2_O molecules adsorbed on the surface of h-WO_3_ nanowire will be oxidized into H^+^ ions and O_2_ by holes injected from the positively-charged electrode or excited by laser illumination. H^+^ ions might accumulate and then transport to the negatively-charged electrode in the condensed water film on the surface of WO_3_ nanowire based on Grotthuss mechanism. The H^+^ ions will cause the effect similar to that of a negative gate voltage and enhance the memristive performance characterized by analogic resistive switching remarkably. When H^+^ ions drift in the lattice of WO_3_ nanowire, conductive H_x_WO_3_ filament might form or break near electrode under bias voltage sweep, which will endow the device with memristive performance characterized by digital resistive switching.

## Results and Discussion

**Figure 1:**
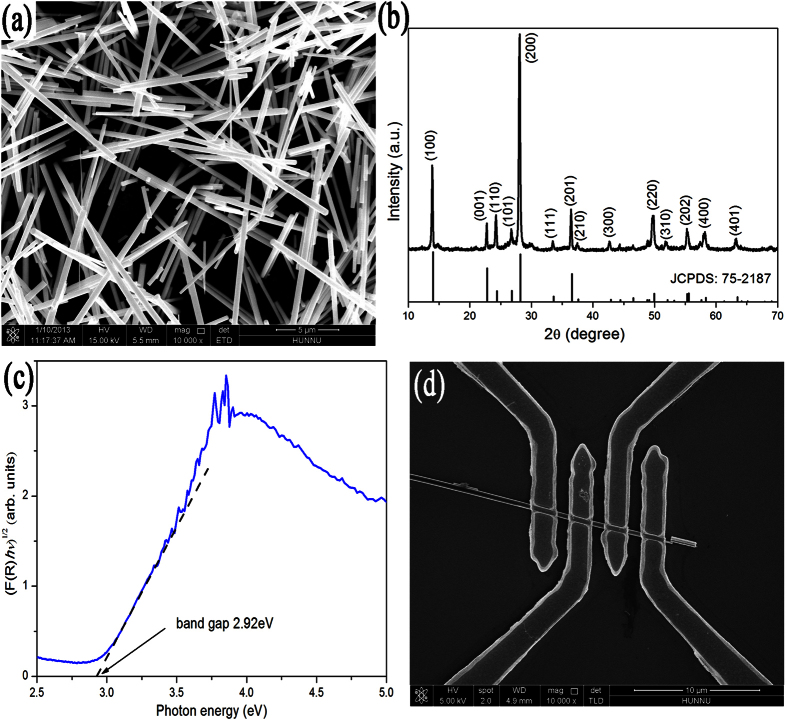
SEM image (**a**), XRD pattern (**b**) and plot of [*F*(*R*)*hν*]^*1*/*2*^ vs photon energy *hν* (**c**) of the as-synthesized WO_3_ nanowires; SEM image (**d**) of the WO_3_ nanowire under Au electrodes for electrical measurements.

Figure 1(a) presents a typical SEM image of the as-synthesized WO_3_ nanowires, which indicates that the nanowires have diameters typically in the range of 100–200 nm and lengths up to ~5 μm. Figure 1(b) displays a typical XRD pattern of the WO_3_ nanowires, which can be perfectly indexed as hexagonal structure of WO_3_ (JCPDS card: 75–2187) without other phases. The sharp diffraction peaks also indicate that the nanowires are well crystallized. In order to obtain the indirect band gap of WO_3_ nanowires, the plots of [*F*(*R*)*hν*]^*1*/*2*^ vs *hν* of WO_3_ nanowires are shown in Fig. 1(c). The *F*(*R*) = (*1 − R*)^*2*^/*2R* = α/*S* is the Kubelka-Munk function, where *R*, *α* and *S* are the diffuse reflection, absorption and scattering coefficient, respectively^33^. The band gap of WO_3_ nanowires can be defined by extrapolating the rising linear part of the plot of [*F*(*R*)*hν*]^*1*/*2*^ vs *hν* to the photon energy axis (dotted line in Fig.1(c)). It indicates that the indirect band gap of WO_3_ nanowires is about 2.92 eV. Figure 1(d) shows a typical SEM image of the Au/h-WO_3_ nanowire/Au device for electrical transport measurements. The spacing between adjacent electrodes is about 1 μm.

**Figure 2:**
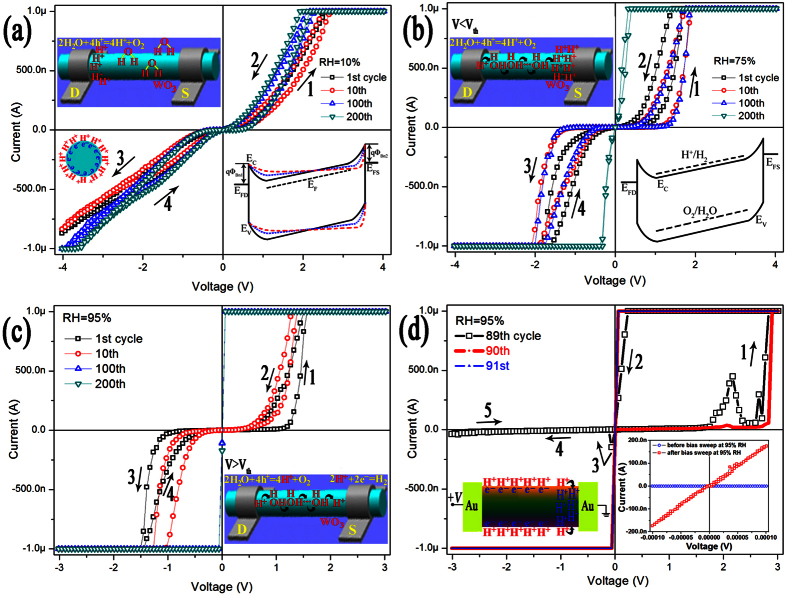
I-V curves of the Au/h-WO_3_ nanowire/Au device recorded repeatedly at 10% RH (**a**), 75% RH (**b**) and 95% RH (**c**), and the three I-V curves displaying the process of insulator-metal transition (**d**). Arrows with number indicating the sweep sequence of bias voltage. The inset of (**a**) band diagram showing the bending of the bands at the Au/WO_3_ interface under a bias (lower right) and schematic diagram showing the distribution of H^+^ ions under lower RH (upper left); The inset of (**b**) band diagram showing energy levels for water oxidation and H^+^ ion reduction (lower right) and schematic diagram showing the distribution of H^+^ ions under higher RH and small bias (upper left); The inset of (**c**) schematic diagram showing H^+^ ions drifting inside the condensed water film through the Grotthuss mechanism under a bias larger than threshold voltage; The inset of (**d**) I-V curves near zero bias recorded before and after 200 cycles of bias sweep at 95% RH (lower right) and schematic diagram showing H^+^ ions being implanted into the lattice of WO_3_ (lower left).

Figure 2 shows the typical current-voltage (I-V) curves of an Au/h-WO_3_ nanowire/Au device recorded repeatedly in pure aqueous vapour with different relative humidity(RH) levels, which indicate that the memristive properties of the device are strongly dependent on the RH level and sweep times of bias voltage. When the RH is about 10%, the I-V curves exhibit noticeable electrical hysteresis with characteristic of “not self-crossing” (Fig. 2(a)), and remain unchanged after 200 cycles of bias voltage sweep. This result shows that the nanowire device has excellent stability in vacuum or at low RH. As shown in the inset of Fig. 2(a), the Au/h-WO_3_ nanowire/Au device can be modeled as being composed of two back-to-back Schottky barriers. As the bias voltage is swept, it falls mainly to the reverse-biased Schottky barrier, and then the electrical field in the barrier might be strong enough for charged ions drifting or carriers self-trapping. Charged ions drifting or carriers self-trapping can lead to the width of the reverse-biased barrier decreasing and the width of the forward-biased barrier increasing, which will touch off resistive switching phenomenon as proposed in previous reports^29^.

While under relatively larger RH of 75% (Fig. 2(b)), I-V curves recorded repeatedly do exhibit different performance. On the one hand, the width of the hysteresis loop of the I-V curve become larger, which indicates that the device exhibits enhanced memristive performance at higher RH level. This result is in accord with what we have previously reported^34^. On the other hand, the electrical hysteresis disappears completely and the conductance of the nanowire increases remarkably after about 200 cycles of bias sweep. It is also indicates that the I-V curves become linear and symmetric near zero bias, which shows that the contacts between the nanowire and Au electrodes are Ohmic contacts, and there is an insulator-metal transition during the bias voltage sweep process. When the device is tested at RH of 95%, the more remarkable increase in conductance and the disappearance of electrical hysteresis can be detected only after about 100 cycles of bias sweep (Fig. 2(c)). The I-V curves recording the process of the insulator-metal transition are shown in Fig. 2(d). The I-V curves under small bias (0.1 mV) recorded before and after the insulator-metal transition (Inset of Fig. 2(d)) show that the resistance (resistivity) of the nanowire at 95% RH can decrease to 550 Ω (1.73 × 10^−3 ^Ω·cm). This insulator-metal transition is closely associated with RH levels, and then cannot be explained only by the formation of an oxygen-deficient conductive filament in the nanowire.

As shown in the band diagram (Inset of Fig. 2(b)), the energy level for water oxidation lies below the top of valence band (for holes) and the energy level for H^+^ ion reduction lies above the bottom of conduction band. Water molecules adsorbed on the surface of WO_3_ can be oxidized to produce H^+^ ions and oxygen gas by holes near the top of the valence band injected from the positively-charged electrode or excited by laser illumination, while H^+^ ions cannot be reduced to produce H_2_ gas by electrons near the bottom of the conduction band^31^. Therefore, there are always H^+^ ions adsorbed on the surface of WO_3_ under ambient atmosphere. At a higher RH (≥51%), there is always a condensed water film on the surface of WO_3_ (at less two physical adsorption water molecule layers)^34^,^35^, and H^+^ ions might diffuse in the water film based on the Grotthuss mechanism.

**Figure 3:**
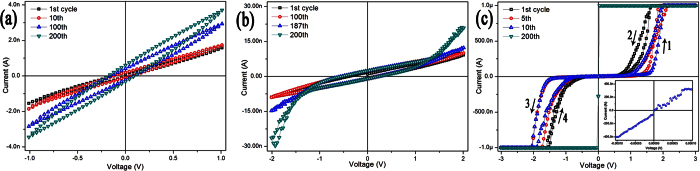
I-V curves of the Au/h-WO_3_ nanowire/Au device recorded repeatedly at 94% RH with bias voltage sweep ranges of ±1 V (**a**), ±2 V (**b**) and ±3 V (**c**). The inset of (**c**) I-V curve near zero bias recorded after 200 cycles of bias sweep with range of ±3 V.

Figure 3 shows I-V curves of the Au/h-WO_3_ nanowire/Au device recorded repeatedly at 94% RH with different bias voltage sweep ranges. Though there are enough water molecules adsorbed on the surface of WO_3_ nanowire at higher RH level (94% RH), the I-V curves recorded repeatedly with bias sweep ranges of ±1 V and ±2 V (Fig. 3(a,b)) don’t differ much from each other, exhibiting no electrical hysteresis and no obvious increase in conductance. However, when recorded repeatedly with bias sweep range of ±3 V at 94% RH (Fig. 3(c)), the I-V curves do exhibit electrical hysteresis and insulator-metal transition after about 200 cycles. The inset of Fig. 3(c) shows that the resistance (resistivity) of the device can decrease to 250 Ω (0.79 × 10^−3^ Ω cm).

Because the enhanced electrical hysteresis of WO_3_ nanowire is closely associated with higher RH (>51%) and larger bias voltage (≥3 V), we have proposed that the enhanced memristive performance can be attributed to H^+^ ions transport in the condensed water film on the surface of the WO_3_ nanowire based on the Grotthuss mechanism^34^. When the bias voltage is applied, H^+^ ions will be generated mainly near the positively-charged electrode due to electrons being majority carriers in n-type semiconducting WO_3_. Under low RH, H^+^ ions might be localized where they are generated and can’t move around. As shown in Fig. 2(a), the H^+^ ions will attract equivalent numbers of electrons in WO_3_ nanowire to form electric double layer and then reduce the concentration of even deplete the majority carriers in WO_3_ nanowire fragment. Because the quantity of H^+^ ions is finite, and the Schottky barrier between the positively-charged electrode and the WO_3_ nanowire is forward biased on the other hand, the presence of the H^+^ ions or the electric double layer has almost no effect on the electrical transport properties of the device. Under high RH, H^+^ ions might exist in the form of H_3_O^+^ in the water film and transport to the negatively-charged electrode based on the Grotthuss mechanism. When the applied bias voltage is small (less than 2 V, for example), the electrons injected from the negatively-charged electrode don’t have enough energy to reduce H^+^ ions, and then the H^+^ ions will accumulate near the negatively-charged electrode under electric field (inset of Fig. 2(b)). The accumulated H^+^ ions will serve as a negative back gate voltage, and the semiconducting nanowire fragment near the negatively-charged electrode will be depleted completely, which will increase the width of the reverse biased Schottky barrier remarkably, and enhance the threshold voltage obviously. When the bias voltage is larger than a critical value (threshold voltage, 2 V), the accumulated H^+^ ions will be reduced by the elevated electrons. H^+^ ions in the water film will drift to the negative electrode and produce a current (Inset of Fig. 2(c)). With the decrease of H^+^ ions in the water film, the majority carriers in the WO_3_ nanowire will increase proportionately, which lead to a significant increase in current. Therefore, the enhanced memristive performance of the Au/h-WO_3_ nanowire/Au device depends on the accumulation and release behavior of H^+^ ions on the surface of WO_3_ nanowire.

All holes injected from the positive-charged electrode can oxidize H_2_O molecules to produce H^+^ ions, but only electrons elevated by applied bias voltage over the energy level of H^+^/H_2_ can eliminate H^+^ ions by reducing them into H_2_. Supposed that the chance of water molecules being oxidized by injected holes is the same as that of H^+^ ions being reduced by injected elevated electrons, there are always a certain number of H^+^ ions left and accumulated on the surface of WO_3_ nanowire in each bias sweep cycle, and then the quantity of H^+^ ions will increase with the number of the bias sweep cycle. H^+^ ions prefer to accumulate at the vicinity of the negative electrode due to the effect of electrical field, and then the more H^+^ ions accumulate, the larger the threshold voltage for the reverse-biased Schottky barrier is. As shown in Fig. 3(c), the threshold voltages for the device increase indeed with the number of bias voltage sweep cycle. Figure 2(d) also indicates that the device is almost blocked completely in the range of bias sweep (the 89^th^ cycle) before the insulator-metal transition occurs. It is reasonable to propose that H^+^ ions can migrate over the surface barrier and into the lattice of WO_3_ nanowire when the concentration of H^+^ ions is high enough. Accordingly, the semiconducting WO_3_ nanowire is converted into metallic-type H_x_WO_3_ nanowire with remarkably enhanced conductance^36^.

**Figure 4:**
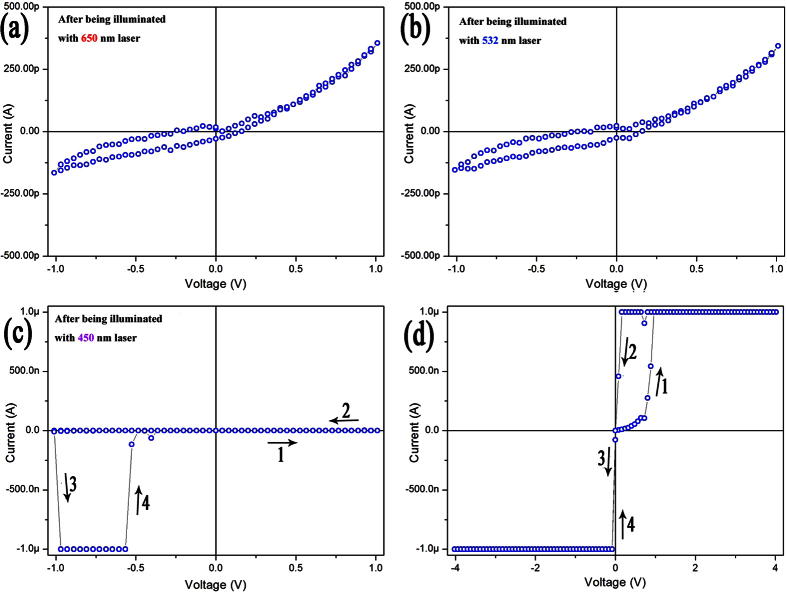
I-V curves of the Au/h-WO_3_ nanowire/Au device recorded after having been illuminated by a continuous laser beam with wavelength of 650 nm (**a**), 532 nm (**b**) and 450 nm (**c**) for 1 minute, 1 minute and 30 seconds, respectively. (**d**) I-V curve recorded with bias voltage sweep range of ±4 V after being illuminated with laser wavelength of 450 nm. Arrows with number indicating the sweep sequence of bias voltage.

Inspired by the photochromism of WO_3_ thin film, we explored herein the effect of light illumination on the memristive performance of the Au/h-WO_3_ nanowire/Au device. Figure 4 shows I-V curves of the device recorded in air (52% RH) after being illuminated by lasers with different wavelengths. It is indicated that illuminations with laser wavelengths of 650 nm and 532 nm have no obvious effect on the electrical transport properties of the device (Fig. 4(a,b)). Figure 4(c) shows that the conductance of the device doesn’t increase immediately under small bias after having been illuminated for 30 seconds with laser wavelengths of 450 nm except for a sudden increase in the current around bias voltage of −1 V, while under relative large bias (±4 V), the device exhibits insulator-metal transition immediately during the first cycle of bias sweep (Fig. 4(d)). The photon energy of 450 nm laser (~2.76 eV) accords with the band gap of WO_3_ nanowires (2.92 eV), which can excite interband transition at room temperature (2.76 eV + 0.26 eV > 2.92 eV). Optically excited holes at the top of valence band will oxidize water molecules adsorbed on the surface of WO_3_ nanowire to produce a large number of H^+^ ions immediately. The H^+^ ions are firstly localized at the surface and can migrate over the surface barrier into the lattice of WO_3_ nanowire quickly under higher bias voltage, and then the semiconducting WO_3_ nanowire is converted into metallic H_x_WO_3_ with remarkably enhanced conductance (Fig. 4(d)).

**Figure 5:**
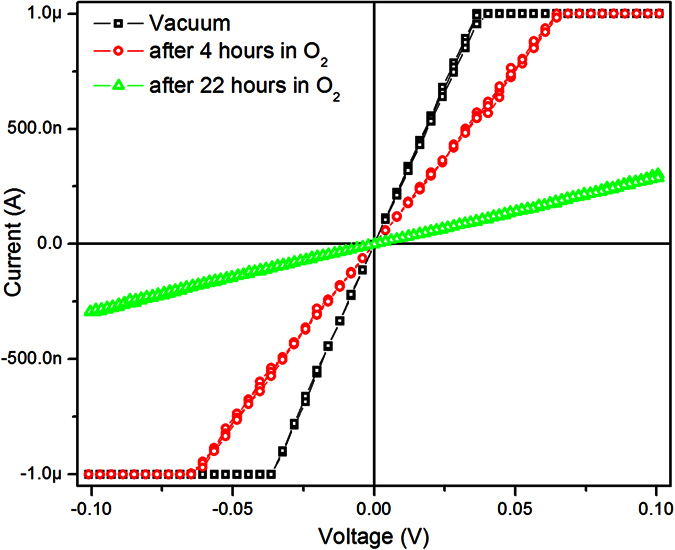
I-V curves of the Au/h-H_x_WO_3_ nanowire/Au device recorded in vacuum and after having been exposed with oxygen atmosphere for different time.

However, even at room temperature H_x_WO_3_ are thermodynamically very unstable towards oxidation^37^. Figure 5 shows I-V curves of the Au/h-H_x_WO_3_ nanowire/Au device recorded in vacuum and in oxygen atmosphere under small bias voltage. Our experimental results (not shown here) prove that the small bias voltage (less than 0.1 V) cannot inject electrons with enough energy to reduce H^+^ ions directly. In vacuum, the nanowire has the resistance of 36.4 kΩ (black square). After having been exposed with O_2_ atmosphere for 4 and 22 hours, the resistance of the device increases to 65.6 kΩ (red circle) and 347 kΩ (green triangle), respectively. The presence of oxygen causes oxidation of hydrogen tungsten bronze, and decreases the concentration of H^+^ ions. The electrical conductivity measurements show that the conductance of the bronze increases as the H^+^ ion concentration gets larger^36^. Therefore, it is possible to modulate the concentration of H^+^ ions in H_x_WO_3_ nanowire simply by exposure with oxygen atmosphere.

**Figure 6:**
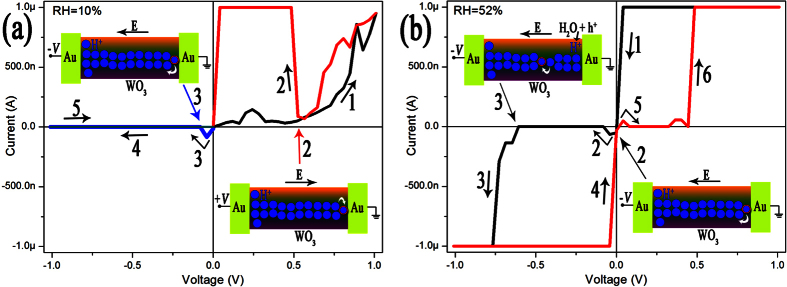
I-V curves of the Au/h-H_x_WO_3_ nanowire/Au device recorded in vacuum (**a**) and in air (**b**) after being standing in oxygen atmosphere for a certain time. Arrows with number indicating the sweep sequence of bias voltage. The inset: schematic diagrams showing H^+^ ions drifting inside the h-WO_3_ nanowire without (**a**) and with (**b**) H^+^ ion supply from water oxidation.

As the standing time in oxygen atmosphere increasing, the concentration of H^+^ ions and then the diameter of H_x_WO_3_ filament in WO_3_ nanowire will decrease. Figure 6 indicates that the Au/h-H_x_WO_3_/Au device exhibits reproducible bipolar resistance switching behavior at low RH (Fig. 6(a)) and unipolar resistance switching behavior at high RH (Fig. 6(b)) after standing in oxygen atmosphere for a certain time. As shown by the schematic diagram (Inset of Fig. 6(a)), the bipolar resistance behavior might be attributed to the formation and rupture of conductive H_x_WO_3_ filament induced by H^+^ ions drifting under different electrical polarity. In the conditions of high RH, it is found that the resistance of the device increases and decreases abruptly under the same electrical polarity. The abrupt increase in resistance near zero bias, just as the case at low RH, can be attributed to the rupture of the conductive filament induced by H^+^ ions drifting. The abrupt decrease in resistance at relative large bias with the same polarity can also be attributed to the formation of conductive H_x_WO_3_ filament. The only difference is that the H^+^ ions for the formation of H_x_WO_3_ filament originate from the oxidation of water molecules absorbed on the surface, not from the H_x_WO_3_ filament itself. After the polarity of bias voltage changes, the H^+^ ions in H_x_WO_3_ filament will drift away from the positive electrode. Although injected holes can oxidize adsorbed water molecules to produce H^+^ ions from the very beginning in the vicinity of positive electrode, as-produced H^+^ ions locate mainly on the surface of the WO_3_ nanowire. Therefore, the conductive H_x_WO_3_ filament will break, and the resistance of the device will increase abruptly. On the other hand, the H^+^ ion depleted nanowire fragment will elevate electric potential of the H^+^ ion on the surface, and help them to drift into the lattice of WO_3_ by overcoming the surface barrier. With the increase of bias voltage, the H^+^ ions will accumulate and form conductive H_x_WO_3_ filament in the depleted nanowire fragment, which lead to an abrupt decrease in resistance of the device.

**Figure 7:**
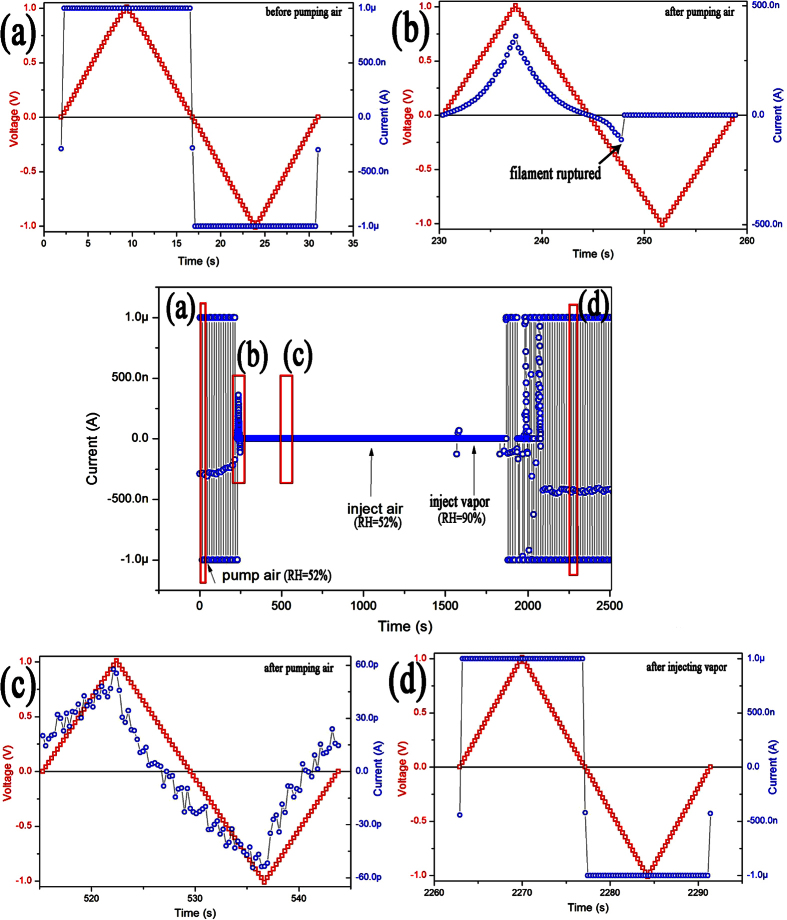
I-V curves of the Au/h-WO_3_ nanowire/Au device recorded continuously in different atmosphere. (**a**) I-V curve of the Au/h-H_x_WO_3_ nanowire/Au device recorded in air (with RH of 52%); (**b**,**c**) I-V curves recorded after air being pumped; (**d**) I-V curve recorded after the RH of the air being elevated over 90%.

In atmosphere with low RH, the dynamic equilibrium of generation, transport and reduction of H^+^ ions will be broken due to lack of water molecules on the surface and continual reductions of H^+^ ions by injected electrons. Figure 7 shows I-V curves of an Au/h-WO_3_ nanowire/Au device recorded continuously in different atmosphere. At the very beginning, stable I-V curves recorded continuously in air with RH of 52% (Fig. 7(a)) indicate that the WO_3_ nanowire has been converted into metallic H_x_WO_3_ nanowire completely. It is also indicates there is always current with certain value (about 250 nA) passing through under zero bias, which suggests that there is an electric potential systematic error (about 47 μV). As the air in test chamber has been pumped out, the current under zero bias decreases gradually at first, and then the current in the range of bias voltage sweeping decreases to dozens of pA immediately (Fig. 7(b,c)). Accordingly, the resistance of the device increases gradually at first, and then increases abruptly, which can correspond to thinning and rupture of the H_x_WO_3_ filament respectively because H^+^ ions are reduced persistently by electrons injected from negative electrode (Fig. 7(b)). When the device is tested in air with RH above 52% again, there are no obvious changes in the recorded I-V curves in short time. Nevertheless, the semiconducting WO_3_ nanowire can be converted into metallic H_x_WO_3_ nanowire again after the bias voltage have been swept in air with RH of 90% for a while (Fig. 7(d)). In Fig. 7, the insulator-metal transition can be realized under bias voltage sweep range of ±1 V, which might be attributed to the Ohmic contacts between the nanowire and electrodes, or only part of the nanowire between electrodes (nanowire fragment) being semiconducting WO_3_.

## Conclusion

The effect of water molecule adsorption on the electrical transport properties of the Au/h-WO_3_ nanowire/Au device has been investigated carefully. I-V curves of a two-terminal Au/h-WO_3_ nanowire/Au device have been recorded repeatedly under different RH levels, with different bias voltage sweep ranges, and after being illuminated by laser with different wavelengths. The experimental results indicate that water-oxidized H^+^ ions might drift on the surface of WO_3_ nanowire based on Grotthuss mechanism when the relative humidity level is high enough, which will enhance the memristive performance characterized by analogic resistive switching remarkably. When the bias voltage is swept persistently under large bias sweep range in high RH level, the H^+^ ions will accumulate gradually in the condensed water film, and immigrate over the surface barrier into the lattice of WO_3_ nanowire, and the semiconducting WO_3_ nanowire can be converted into metallic H_x_WO_3_ nanowire gradually. The accumulation of H^+^ ions can be realized more easily after being illuminated with 450 nm laser illumination. By modulating the concentration of hydrogen ions, conductive hydrogen tungsten bronze filament might form or break near electrodes when the polarity of applied voltage changes during a cycle of bias sweep, which will endow the device with memristive performance characterized by digital resistive switching.

## Methods

### Nanowires synthesis

The h-WO_3_ nanowires used in this investigation were synthesized using a simple hydrothermal method as previously reported^30^. In a typical synthesis, 8.25 g sodium tungstate (Na_2_WO_4_·2H_2_O) was dissolved in 250 mL deionized water. Hydrochloric acid (HCl, 3 M) was used to adjust the PH value of the Na_2_WO_4_ solution to 1.2. After being filtered, the precipitate was washed sequentially with deionized water and ethanol to remove contaminant ions, and then dispersed in 200 mL citric acid (C_6_H_8_O_7_, 0.1 M) to form a translucent homogeneous and stable WO_3_ sol. A 45 mL volume of WO_3_ sol was transferred into a 50 mL autoclave, and then 1.3 g potassium sulfate (K_2_SO_4_,) was added to the sol. The autoclave was sealed and maintained at 240 °C for 32 h, and then cooled down to room temperature. The precipitates in the solution were filtered, washed sequentially with deionized water and ethanol to remove possible remnantions, and then dried at 60 °C.

### Material Characterizations

The morphologies of the final product were characterized on a scanning electron microscope (SEM, FEI, NovaSEM-450). The crystal structure was determined by x-ray diffraction (XRD, Aolong Y2000, Cu Kα, λ = 0.15405 nm). The absorption spectra were characterized by an UV/Vis/NIR spectrophotometer equipped with a 60 mm integrating sphere (Perkin Elmer Lambda 750).

### Device Fabrication

The individual WO_3_ nanowire based devices were fabricated on heavily n-doped Si substrate covered with a 100 nm thick thermally grown SiO_2_ layer. Electrodes were defined on the top of the WO_3_ nanowires by using a standard photolithography technique, and formed by metal deposition (100 nm thick Au) and a lift-offprocess.

### Electrical Measurement

Electrical transport measurements were conducted on a probe station at room temperature by using a semiconductor characterization system (Keithley 2602). The probe station is placed in a homemade vacuum chamber, which is firstly vacuumized to a base pressure less than 10^−1^ Pa by mechanical pump and then filled with water vapor through an injector. The relative humidity in the chamber can be adjusted simply by vacuumizing and water evaporating. The accuracy of the humidity sensor used in our experiments is about ±1%. In order to avoid devices being destroyed by Joule heat, the maximum current flowing through the nanowire is limited at 1000 nA.

## Additional Information

**How to cite this article**: Zhou, Y. *et al*. Modulating memristive performance of hexagonal WO_3_ nanowire by water-oxidized hydrogen ion implantation. *Sci. Rep*. **6**, 32712; doi: 10.1038/srep32712 (2016).
